# The Alpha-Synuclein Gene (*SNCA*) is a Genomic Target of Methyl-CpG Binding Protein 2 (MeCP2)—Implications for Parkinson’s Disease and Rett Syndrome

**DOI:** 10.1007/s12035-024-03974-3

**Published:** 2024-03-02

**Authors:** Ina Schmitt, Bernd O. Evert, Amit Sharma, Hassan Khazneh, Chris Murgatroyd, Ullrich Wüllner

**Affiliations:** 1 Department of Neurology, University of Bonn, Bonn, Germany; 2 German Centre for Neurodegenerative Disease (DZNE), Bonn, Germany; 3 Department of Neurosurgery, University of Bonn, Bonn, Germany; 4 Department of Life Sciences, Manchester Metropolitan University, Manchester, UK; 5 Department of Neurodegenerative Diseases, University of Bonn, Bonn, Germany

**Keywords:** DNA methylation, Epigenetic, Alpha-synuclein, *SNCA*, Intron, Methyl-CpG binding protein 2, MeCP2, Genomic target, Parkinson’s disease, Rett syndrome, RTT

## Abstract

Mounting evidence suggests a prominent role for alpha-synuclein (a-syn) in neuronal cell function. Alterations in the levels of cellular a-syn have been hypothesized to play a critical role in the development of Parkinson’s disease (PD); however, mechanisms that control expression of the gene for a-syn (*SNCA*) in *cis* and *trans* as well as turnover of a-syn are not well understood. We analyzed whether methyl-CpG binding protein 2 (MeCP2), a protein that specifically binds methylated DNA, thus regulating transcription, binds at predicted binding sites in intron 1 of the *SNCA* gene and regulates a-syn protein expression*.* Chromatin immunoprecipitation (ChIP) and electrophoretic mobility-shift assays (EMSA) were used to confirm binding of MeCP2 to regulatory regions of *SNCA*. Site-specific methylation and introduction of localized mutations by CRISPR/Cas9 were used to investigate the binding properties of MeCP2 in human SK-N-SH neuroblastoma cells. The significance of MeCP2 for *SNCA* regulation was further investigated by overexpressing MeCP2 and mutated variants of MeCP2 in *MeCP2* knockout cells. We found that methylation-dependent binding of MeCP2 at a restricted region of intron 1 of *SNCA* had a significant impact on the production of a-syn. A single nucleotide substitution near to CpG1 strongly increased the binding of MeCP2 to intron 1 of *SNCA* and decreased a-syn protein expression by 60%. In contrast, deletion of a single nucleotide closed to CpG2 led to reduced binding of MeCP2 and significantly increased a-syn levels. In accordance, knockout of *MeCP2* in SK-N-SH cells resulted in a significant increase in a-syn production, demonstrating that *SNCA* is a genomic target for MeCP2 regulation. In addition, the expression of two mutated MeCP2 variants found in Rett syndrome (RTT) showed a loss of their ability to reduce a-syn expression. This study demonstrates that methylation of CpGs and binding of MeCP2 to intron 1 of the *SNCA* gene plays an important role in the control of a-syn expression. In addition, the changes in *SNCA* regulation found by expression of MeCP2 variants carrying mutations found in RTT patients may be of importance for the elucidation of a new molecular pathway in RTT, a rare neurological disorder caused by mutations in *MECP2*.

**Supplementary Information:**

The online version contains supplementary material available at 10.1007/s12035-024-03974-3.

## Background

Alpha-synuclein (a-syn) is an abundant protein in mammalian neurons and numerous cells in the hematopoietic lineage. It has been implicated in various cellular functions, and the altered expression of the gene for a-syn (*SNCA*) has profound effects on many intracellular processes [1]. a-syn is a major component of Lewy bodies, the pathological hallmark found in both familial and sporadic Parkinson’s disease (PD) patients [2, 3]. In kindreds with *SNCA* multiplication and familial parkinsonism, increased a-syn dose-dependently results in severe parkinsonian phenotypes [4]. Several studies have identified significant risk haplotypes for sporadic PD (sPD) that are predicted to regulate *SNCA* expression (PDbase; [5]). Extensive promoter analyses and studies of *SNCA* mRNA in dopaminergic neurons (DN) showed that genotype-dependent regulatory mechanisms of *SNCA* expression contribute to the risk of sPD, and a recent study revealed that its expression in brain *in vivo* is regulated predominantly by intronic enhancers [6–9]. Previous studies have independently identified intron 1 as a regulatory region of *SNCA* with expression-relevant GATA binding sites and NGF response elements in intron 1 of rodent *Snca* [10, 11] and binding of ZSCAN21 to human *SNCA* intron 1 [12]. We have shown previously that methylation of *SNCA*_(-926/-483)_ in intron 1 of the *SNCA* gene is decreased in sPD patients’ brains and represses expression of *SNCA*, which was confirmed in human iPSC-derived neurons by targeted editing of DNA methylation of *SNCA* intron 1 [13, 14]. The importance of DNA methylation for a variety of conditions and functions of the central nervous system (CNS) in addition to its pivotal role in malignancies has been well demonstrated [15]. Depending on the methylation status of CpGs within gene regulatory regions, methyl-CpG-binding domain (MBD) proteins can differentially bind and recruit co-repressor complexes to mediate transcriptional repression by a conformational change of the chromatin structure [16, 17]. Some transcription factors (TF) may also bind their regulatory elements in a methylation-dependent manner [18].

Several putative TF binding site motifs are found within a region of 23 CpG dinucleotides within *SNCA*_(-926/-483)_, including methyl-CpG binding protein 2 (MeCP2) binding motifs [9, 14]. MeCP2 is essential for the normal function of neurons and is a key regulator of gene transcription—it has been suggested that MeCP2 may bind to more than half of all promoters of genes expressed in the CNS [19, 20]. MeCP2 has been shown to bind to methylated CpGs with A/T sequence stretches in the vicinity [21]—although the precise identity of all target sequence motifs has not yet been fully elucidated [22]. In addition, hydration of methylated DNA [23], non-CG context [24], and DNA sequence properties in *cis* [25] are also important factors for the binding of MeCP2 to DNA sequences. Missense or nonsense mutations in the *MeCP2* gene have been linked to the etiology of Rett syndrome (RTT), a major cause of mental retardation in girls with a prevalence of about 1 in 10,000 female births [26–28]. The *MeCP2* gene is located on the X chromosome, resulting in a mosaic of wild-type and mutant MeCP2-expressing cells in women due to X chromosome inactivation. Though all *MeCP2* mutations were initially thought to be lethal in males, increasing numbers have been identified recently in around 1.3 to 1.7% of males with mental retardation [29]. Different *MeCP2* mutations lead to a wide spectrum of neurological disorders, ranging from mild mental retardation to severe neonatal encephalopathy [30]. Parkinsonism is a particularly frequent symptom of RTT and *MeCP2* mutations [31].

Several observations point to a mechanistic link between pathophysiological characteristics observed in PD and RTT. For example, analyses of cerebrospinal fluid from RTT patients have found reduced levels of dopamine [32, 33], while another study showed a change in dopaminergic neuron (DN) metabolism related to abnormal motor movements and late motor deterioration [34]. Changes in dopamine D2 receptors in the basal ganglia have also been reported [35, 36]. Neuropathological studies support an involvement of the nigrostriatal system in RTT with a reduction in the number of DN in the substantia nigra [37] and corresponding reduction of tyrosine hydroxylase (TH) immunoreactivity [38] and an abnormal thinning of dendrites in the substantia nigra [39]. A *Mecp2* knockout (ko) mouse model, carrying a deletion of *Mecp2* exons 3 and 4, showed a strong reduction in TH-expressing DN, while treatment with levodopa and a DOPA-decarboxylase inhibitor, the gold standard treatment for PD patients, markedly improved the associated behavioral abnormalities [40, 41]. In another model of *Mecp2*-null mice, levels of norepinephrine and dopamine were reduced [42] and TH-expressing DN diminished [43]. Taken together, these data strongly suggest that genes implicated in the pathophysiology of PD are among the target genes of MeCP2.

Here, we provide evidence for methylation-dependent binding of MeCP2 to a restricted region of *SNCA* intron 1. The effects of *MeCP2* knockout and overexpression of variants carrying the four common mutations causing RTT on a-syn levels in addition suggest that *SNCA* regulation could be impaired in some RTT patients.

## Materials and Methods

### Cell Culture

SK-N-SH and HeLa cells were cultured in DMEM (Millipore) and RPMI 1640 (PAA Laboratories), respectively, supplemented with 10% fetal bovine serum (FBS Gold, PAA Laboratories), 100 U/ml penicillin, and 100 μg/ml streptomycin (PAA Laboratories) at 37 °C and 5% CO_2_.

For treatment with 5-aza-2′-desoxycytidin (Aza), 5 × 10^6^ SK-N-SH cells were seeded in 10-cm plates, cultured overnight and treated with 10 μM Aza (A3656, Sigma; dissolved in DMSO) or with DMSO as control. The treatment was repeated (including medium replacement) every 12 h for 48 h. The methylation levels of *SNCA*_(-926/-483)_ (CpGs 1-23) were controlled by bisulfite sequencing as previously described [13].

### Human Cortex Samples

Cortex samples from two PD patients and two healthy controls (males, mean age 74.5 ± 2.5, post mortem time 25.3 ± 2.3 h) were obtained from the GermanBrainNet (Ethical approval: 051/00 and 078/20).

### ChIP

For 10 independent chromatin immunoprecipitation (ChIP) experiments, 300-mg cortex or 1 × 10^7^ SK-N-SH cells were cross-linked by 1% formaldehyde and the tissue disaggregated using a Dounce homogenizer. After cell lysis, chromatin was sheared by sonication, and DNA was purified using the Magna ChIP G kit (Millipore) according to the manufacturer’s instruction. The antibody against MeCP2 was kindly provided by C.M. and previously described [44].

### qPCR

Quantitative PCR (qPCR) was performed in triplicate with SYBR Green JumpStart Taq ReadyMix (Sigma) on an Applied Biosystems 7500 Fast Real-Time PCR System using 2 μl of ChIP purified DNA for each reaction. Results were normalized for IgG binding and related to the input DNA. Data are presented as means ± s.d. of three independent ChIP reactions. All primers used in the study are listed in Table 1.

**Table 1:** Primer sequences

Name	Position	Use	Sequence
SYN-F1	SNCA, Intron1	BS-PCR	GGAGTTTAAGGAAAGAGATTTGATT
SYN-R2	SNCA, Intron1	BS-PCR	CAAACAACAAACCCAAATATAATAA
SNCA-CHIP-1F	SNCA, Intron1	qPCR, PCR	GGGCCAGGTCTCTGGGAGGTG
SNCA-CHIP-1R	SNCA, Intron1	qPCR	CGCTCCATGGAGCATCCTCG
SNCA-CHIP-2F	SNCA, Intron1	qPCR	CGAGGATGCTCCATGGAGCG
SNCA-CHIP-2R	SNCA, Intron1	qPCR, PCR	CAGCCTCCACCCTAGCGGACC
SNCA_CpG1-F	SNCA, Intron1	GE	GAGAAGGGAATATCAGAAGCGTTTT
SNCA_CpG1-R	SNCA, Intron1	GE	GCTTCTGATATTCCCTTCTCCGGTG
SNCA_CpG2-F	SNCA, Intron1	GE	AGAGATTAGGCTGCTTCTCCGTTTT
SNCA_CpG2-R	SNCA, Intron1	GE	GGAGAAGCAGCCTAATCTCTCGGTG
SNCA-1274F	SNCA, Intron1	PCR, Seq	GAGAACGCCGGATGGGAGAC
SNCA-1751R	SNCA, Intron1	PCR, Seq	CTCACACTCGCGGGCCGTC
MeCP2_TS1-F	MeCP2, Exon4	GE	GGAGGCTCACTGGAGAGCGAGTTTT
MeCP2_TS1-R	MeCP2, Exon4	GE	TCGCTCTCCAGTGAGCCTCCCGGTG
MeCP2_GCD1-F	MeCP2, Exon4	PCR, Seq	CATCACCACCACTCAGAGTCCC
MeCP2_GCD1-R	MeCP2, Exon4	PCR, Seq	GTGTTTGTACTTTTCTGCGGC
MeCP2_TS3-F	MeCP2,Exon3	GE	CATCATACTTCCCAGCAGAGCGGGTTTT
MeCP2_TS3-R	MeCP2,Exon3	GE	CCGCTCTGCTGGGAAGTATGATGCGGTG
MeCP2_TS4-F	MeCP2,Exon2	GE	CCATGGAATCCTGTTGGAGCTGGGTTTT
MeCP2_TS4-R	MeCP2,Exon2	GE	CCAGCTCCAACAGGATTCCATGGCGGTG
RV3	pGL4.23	qPCR	TAGCAAAATAGGCTGTCCCC
RV3-Rev	pGL4.23	qPCR	AACAGTACCGGATTGCCAAG
SNCA-EMSA-1F	SNCA, Intron1	EMSA	CTGGCTTTCGTCCTGCTTCTGATATTCCCTTCTC
SNCA-EMSA-1R	SNCA, Intron1	EMSA	GAGAAGGGAATATCAGAAGCAGGACGAAAGCCAGCCAGACCAGGGCAC
SNCA-EMSA-2F	SNCA, Intron1	EMSA	GCTGAGAGATTAGGCTGCTTCTCCGGGATCCGC
SNCA-EMSA-2R	SNCA, Intron1	EMSA	GCGGATCCCGGAGAAGCAGCCTAATCTCTCAGCCCAGACCAGGGCAC
LUEGO	/	EMSA	IRDye700-GTGCCCTGGTCTGG

*ChIP* chromatin immunoprecipitation, *(q-) PCR* (quantitative-) polymerase chain reaction, *GE* genome editing, *Seq* sequencing, *EMSA* electrophoretic mobility-shift assay

### Analysis of DNA Methylation and In Vitro Methylation

To eliminate certain recognition sites for restriction enzymes in the vector backbone, the insert from the previously described *SNCA*_(-1524/-189)_ reporter construct [13] was subcloned into the plasmid pUHC-13-3 [45] after removal of the intrinsic CMV promoter. An intrinsic HpaI restriction site was removed by *in vitro* mutagenesis. For site-specific methylation (Fig. 3A), two different fragments were excised by digestion with BstXI/BamHI and BamHI/HpaI, respectively. After *in vitro* methylation with M.SssI and S-adenosylmethionine (New England Biolabs), the methylated fragments were re-ligated into the vector construct, transfected into HeLa cells, and luciferase activity was compared to the respective unmethylated fragment. The extent of methylation in the vector constructs and its conservation after transfection was controlled by bisulfite sequencing as previously described [13].

### Luciferase Reporter Assay

HeLa cells (1 × 10^6^ per well) were seeded in 24-well plates and transfected with the indicated luciferase reporter constructs w/o *in vitro* methylation using Lipofectamine 2000 Reagent (Invitrogen) according to the instructions of the manufacturer. Renilla luciferase (pRL-CMV, Promega) was cotransfected to normalize for transfection efficiency. Cells were harvested 24 h after transfection, and luciferase activities were measured using the Dual Luciferase Reporter Assay System (Promega) in a Centro LB 960 luminometer (Berthold Technologies).

### Genome Editing

CRISPR/Cas9 constructs were generated by insertion of double-stranded oligonucleotides (Table 1) into the GeneArt CRISPR nuclease (OFP reporter) vector (Life Technologies) according to the manual. SK-N-SH cells (1 × 10^6^ per dish) were seeded on 10-cm dishes and transfected with 10 μg of the indicated CRISPR constructs each with 2 μg of pPUR vector (Clontech) using 50 μl ROTIFect PLUS (Carl Roth) according to the instructions of the manufacturer. Two days after transfection, medium was supplemented with 2 μg/ml puromycin (Sigma). Individual resistant clones were selected for 3 weeks and transferred into single wells of a 24-well plate. Cell clones were then analyzed for expression changes of the targeted gene in Western blot analyses. CRISPR/Cas9-mediated localized mutations within *SNCA* intron 1 were analyzed by sequencing of correspondingly generated PCR products (primers are listed in Table 1).

### Western blot analysis

Protein extracts were prepared by resuspending cells in lysis buffer (50 mM Tris–HCl, pH 8.0, 150 mM NaCl, 0.5% Triton × 100, 10 mM MgCl_2_, 1× Halt protease inhibitor-cocktail (Thermo Fisher), 1 μl/ml Benzonase (Thermo Fisher). After incubation on ice for 1 h, 50 μg of protein per lane was separated by SDS gel electrophoresis, transferred to nitrocellulose, and detection was performed with the ECL Western blotting detection system (GE Healthcare) and secondary antibodies conjugated with horseradish peroxidase. The following antibodies and dilutions were used: anti-a-syn, 1:2000 (610786, BD Bioscience); anti-MeCP2, 1:1000 (ab2828, Abcam); anti beta-actin, 1:10000 (A5441, Sigma); goat anti-mouse, 1:2000 (P044701-2, DAKO); and goat anti-rabbit, 1:3000 (7074, Cell Signaling). Immunoreactive signals were detected using the Intas ChemoCam system (Intas) and the software ChemoStar (Intas). Quantification of the signal intensities was performed using ImageJ.

### Protein Expression

For the generation of MeCP2 variants, mutations described in RTT patients (27) were inserted into a *MeCP2* expression plasmid (pCMV3-MeCP2, isoform 1; Sino Biological Inc.) by in vitro mutagenesis.

For transfection, 5 × 10^5^ wild type (wt) or *MeCP2* knockout SK-N-SH cells per well were seeded in six-well plates and transfected with 1-μg expression plasmids using 2.5-μl ROTIFect PLUS (Carl Roth) according to the instructions of the manufacturer. Cells were harvested 24 h after transfection, and lysates prepared for Western blot analysis.

### Electrophoretic mobility-shift assay (EMSA)

Double-stranded (ds) DNA probes were prepared with IRDye700-labeled LUEGO oligonucleotides according to [46] in a ratio of 3:3:1 (LUEGO: forward primer: reverse primer; Table 1). Naïve or *in vitro* methylated probes were cleaned with Oligo Clean & Concentrator kit (Zymo Research).

*MeCP2* cDNA was cloned w/o mutations in pET30b (Novagen). Recombinant His-MeCP2 fusion proteins were generated in *E. coli* BL21, purified by His-spin protein miniprep according to the instructions of the manufacturer (Zymo Research) and quantified with NanoDrop One (Thermo Scientific). For electrophoretic mobility shift assays (EMSAs), 50 fmol labeled ds DNA probes were incubated with 30-75 nmol MeCP2 in 5-μl binding buffer (10 mM Tris, pH 7.5, 1 mM EDTA, 100 mM KCl, 100 μM DTT, 5 % glycerol, 10 μg/ml BSA) for 30 min at room temperature. The binding reactions were separated in 5% non-denaturing polyacrylamide gels. After pre-running the gels without samples for 30 min at 55 V, the samples were finally separated at 55 V for 110 min in 1× TAE buffer. Fluorescence was measured on a LI-COR Odyssey CLx using the Image Studio software (LI-COR Biosciences). Signal intensities were calculated using ImageJ.

### Statistical Analysis

Statistical analysis was performed from at least three individual experiments. Data are expressed as the mean ± s.d. (standard deviation). All data were analyzed by one-way analysis of variance (ANOVA). A *p*-value ≤ 0.05 was considered significant (*p*-value ≤ 0.05 (*), *p*-value ≤ 0.01 (**)).

## Results

### Potential MeCP2 Binding Sites in Intron 1 of the SNCA Gene

**Fig. 1.**
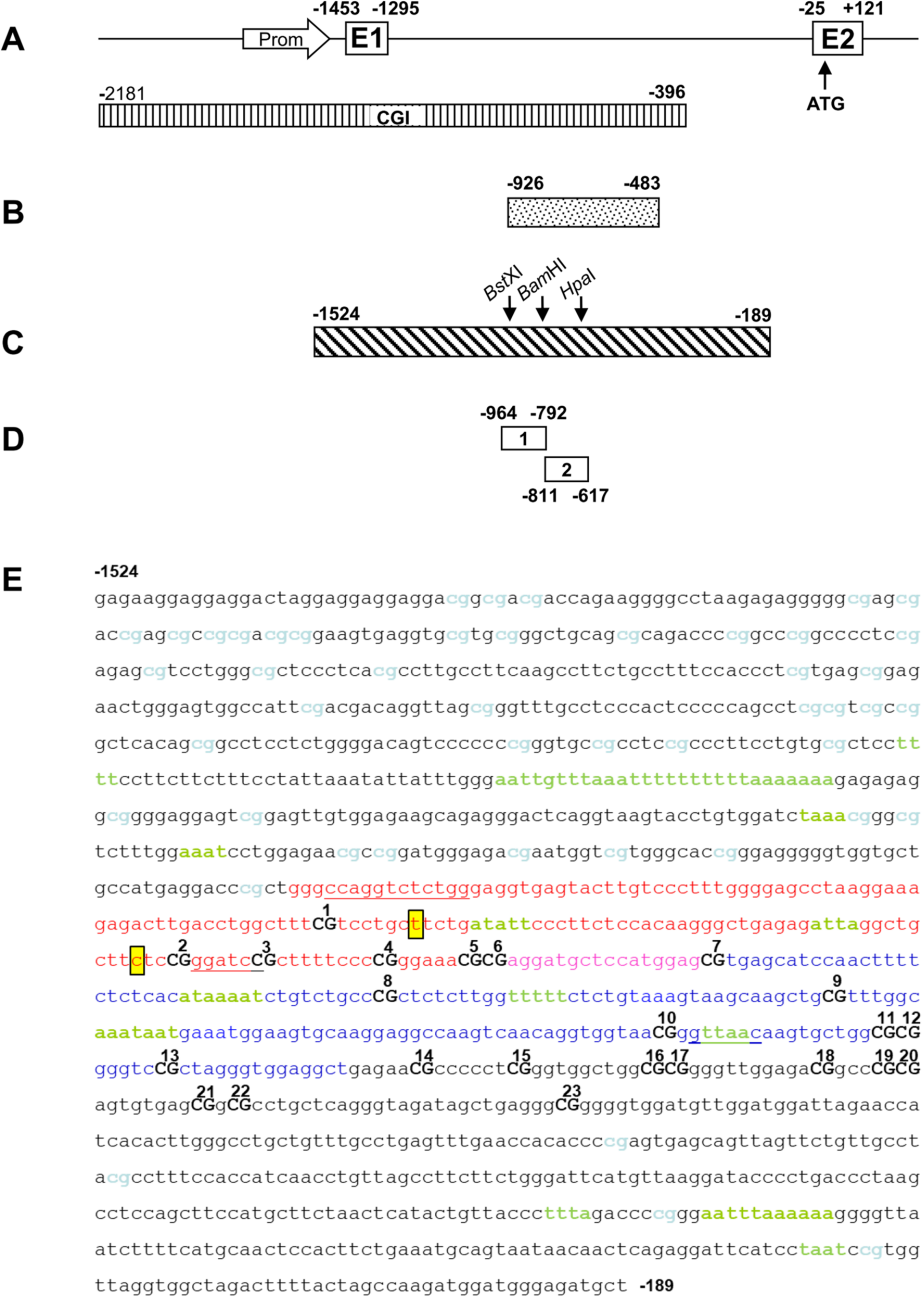
*SNCA* gene regions analyzed for DNA methylation and MeCP2 binding. **A** Schematic of the *SNCA* promoter (arrow) and exons 1 (E1) and 2 (E2). Position of the CpG island (CGI) is depicted by the striped box (adapted from 13). **B** Position of the *SNCA*_(-926/-483)_ region containing CpGs 1-23 analyzed by bisulfite sequencing. **C** Position of the *SNCA*_(-1524/-189)_ region used for reporter assays. Arrows indicate the position of restriction sites for the enzymes BstXI, BamHI, and HpaI. **D** PCR-amplified regions within *SNCA* intron 1 analyzed by ChIP assays with an MeCP2 antibody (ChIP1, -964/-792; ChIP2, -811/-617). **E** Sequence of *SNCA*_(-1524/-189)_ showing CpGs in blue, CpGs 1-23 as part of *SNCA*_(-926/-483)_ in black bold capitals. Sequence of ChIP1 (CpGs 1-7) is marked in red and pink, sequence of ChIP2 (CpGs 6-13) in pink and blue letters. [A/T] ≥ 4 motifs adjacent to CpGs are marked in green; recognition sites for the restriction enzymes BstXI, BamHI, and HpaI are underlined, and target sequences for CRISPR/Cas9 editing are highlighted in yellow boxes

DNA methylation of *SNCA* intron 1 has been shown to repress *SNCA* gene expression whereas demethylation increases *SNCA* expression [14, 47]. Methylated CpGs generate binding sites for proteins containing a methyl-CpG binding domain such as MeCP2 and are associated with transcriptional repression and chromatin remodeling [22]. Thus, we asked whether methylated CpGs in the subregion of *SNCA* intron 1 could be targets for MeCP2 and mediate downregulation of *SNCA* transcription. For efficient binding, MeCP2 requires an A/T-rich sequence flanked by a methylated CpG [22]. In the CpG island of *SNCA* intron 1 (region from − 926/− 483, Fig. 1A, B), five potential MeCP2 binding sites are found in close proximity to CpGs 1, 2, 8, 9, and 10 (Fig. 1E).

### MeCP2 Binds to Methylated CpGs in SCNA Intron 1

**Fig. 2:**
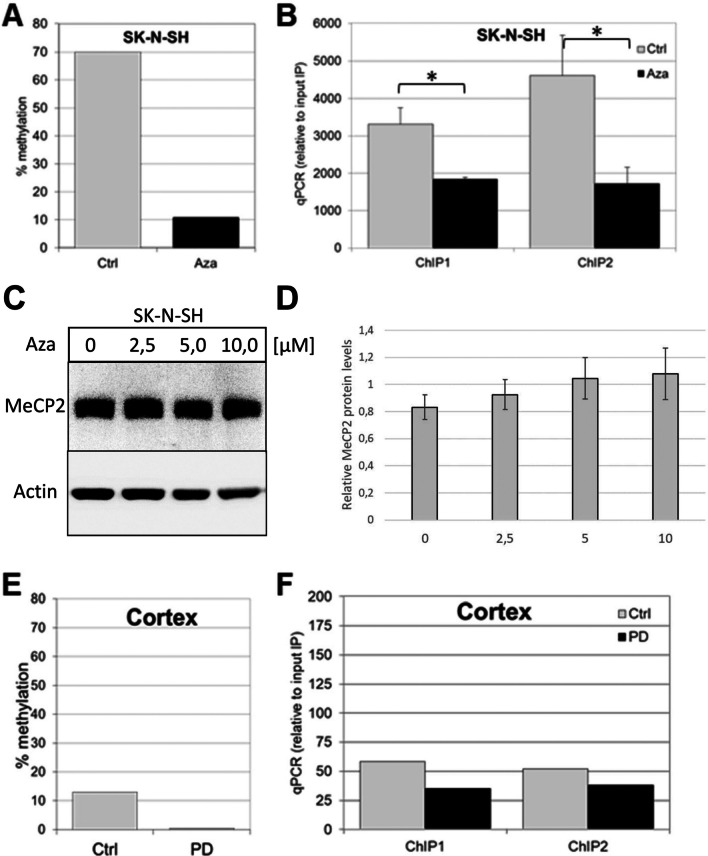
Impact of *SNCA* methylation on MeCP2 binding. **A** Methylation status of *SNCA*_(-926/-483)_ in SK-N-SH cells untreated (Ctrl) and treated with 10 μM Aza for 48 h (Aza). The degree of methylation is presented as the average percentage determined from 10 independent clones (*n* = 10; ± s.d.) by bisulfite sequencing in either the untreated and treated SK-N-SH cells. **B** Enrichment of *SNCA* fragments ChIP1 and ChIP2 for MeCP2 binding in Ctrl and Aza-treated SK-N-SH cells. Data are presented as means ± s.d. of three independent ChIP reactions. **C** Representative Western blot analysis of MeCP2 and actin expression in SK-N-SH cells treated with Aza (0, 2.5, 5.0, and 10.0 μM) for 48 h. **D** Densitometric quantification of the Western blot results for MeCP2 protein levels. The values are expressed in relation to the respective actin levels. The results were averaged from triplicates of three independent experiments and are presented as the mean ± s.d. **E** Methylation status of *SNCA* intron 1 in human brain cortex from two healthy control (Ctrl) and two PD cases (PD). **F** MeCP2 binding to *SNCA* fragments ChIP1 and ChIP2 using ChIP analysis of human cortex samples from two controls (Ctrl) and 2 PD patients (PD)

To verify if these putative CpGs are bound by MeCP2, we performed chromatin immunoprecipitation (ChIP) analyses with a MeCP2-specific antibody and analyzed binding of MeCP2 to two different subregions of *SNCA* intron 1 containing CpGs 1-7 (ChIP1) and CpGs 6-13 (ChIP2) by qPCR (Fig. 1D, E). ChIP analyses were performed using chromatin DNA isolated from SK-N-SH cells that were either treated with the demethylating agent 5-aza-2′-deoxycytidine (Aza) or left untreated. Treatment with Aza decreased methylation levels in *SNCA* intron 1 (− 926/− 483) by approximately 60% compared to untreated control cells (Fig. 2A). Subsequent ChIP assays of chromatin from untreated cells revealed a strong enrichment of MeCP2 in both analyzed regions of the *SNCA* intron 1, ChIP1 and ChIP2 (Fig. 2B). In contrast, ChIPs using chromatin from demethylated, Aza-treated cells revealed a significantly decreased binding of MeCP2 by 50 to 60% for the ChIP1 and ChIP2 regions (*p* = 0.01 and 0.04), respectively (Fig. 2B). Verification of the endogenous MeCP2 levels in Aza-treated SK-N-SH cells showed that decreased binding of MeCP2 to ChIP1 and ChIP2 was not due to a reduction of endogenous MeCP2 expression in Aza-treated cells (Fig. 2C, D). Thus, MeCP2 binds to the *SNCA* intron 1 region, and binding of MeCP2 depends on the degree of DNA methylation.

To evaluate MeCP2 binding to *SNCA* intron 1 in human brain tissue, we prepared DNA and chromatin from different human cortex samples and performed bisulfite sequencing and ChIP assays (*n* = 2 healthy controls, *n* = 2 PD patients). Sequencing of bisulfite converted DNA revealed far lower levels of DNA methylation within *SNCA* intron 1 in PD (0.4% ± 0.1%) compared to healthy controls (13.1% ± 0.6%), though no statistical analyses were performed due to the low group numbers (Fig. 2E). ChIP assays of the subregions in *SNCA* intron 1, ChIP1 and ChIP2, showed a trend towards reduced binding of MeCP2 in PD compared to healthy controls, but again, the small group numbers prevented statistical analysis (Fig. 2F).

### Functional Impact of Methylated CpGs on SNCA Gene Transcription

**Fig. 3:**
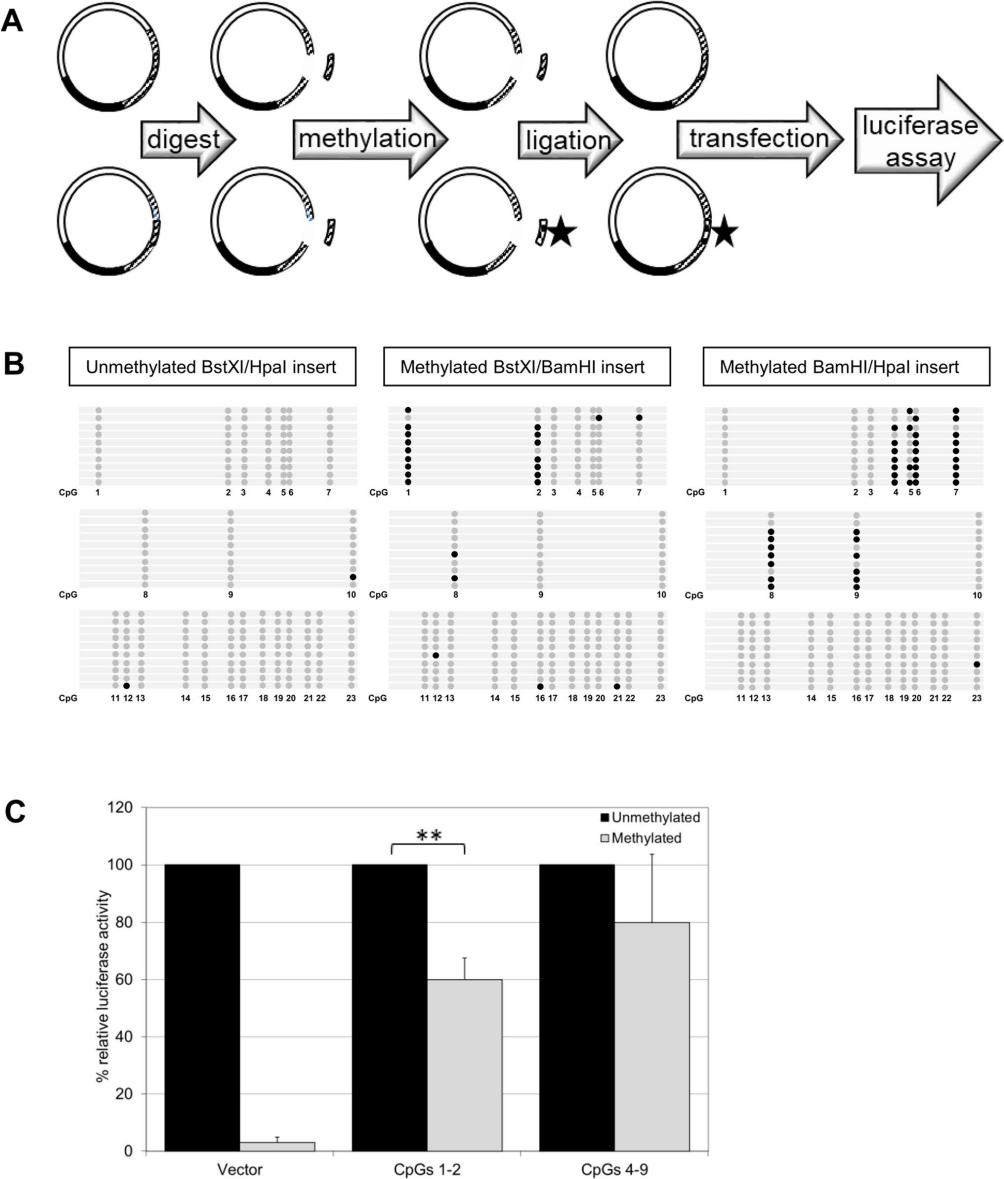
**A** Schematic representation of site-specific methylation (asterisk); for details see “Materials and Methods”. **B** Degree of *SNCA* methylation in the vector construct after transfection into HeLa cells; 10 independent clones were used for bisulfite sequencing of *SNCA*_(-926/-483)_. The lollipop diagram illustrates unmethylated CpGs by open circles and methylated CpGs by closed circles. Left: unmethylated BstXI/HpaI fragment. Middle: site-specific methylation of the BstXI/BamHI fragment. Right: site-specific methylation of the BamHI/HpaI fragment. **C** Luciferase activity after *in vitro* methylation of the entire *SNCA*_(-1524/-189)_ construct (vector) and after site-specific methylation of the BstXI/BamHI fragment (CpGs 1-2) and the BamHI/HpaI-fragment (CpGs 4-9). Data are presented as means ± s.d. of six independent luciferase measurements. (*) *p* ≤ 0.05; (**) *p* ≤ 0.01

To evaluate the functional effects of methylation of individual CpG sites on *SNCA* gene expression, two *SNCA* intron 1 fragments, one containing CpGs 1-2 (BstXI/BamHI fragment) and another one containing CpGs 3-10 (BamHI/HpaI fragment) (Fig. 1C), were subcloned in luciferase reporter constructs (Fig. 3A). Based on these constructs, two additional reporter constructs were generated with the same inserts but with methylated CpGs. For this purpose, the inserts were excised from the above reporter constructs and methylated in vitro using a CpG DNA methyltransferase. The methylated inserts were re-inserted into the non-methylated reporter constructs to ensure that only the CpGs of the insert were methylated. Analysis of the methylation levels revealed increased methylation of CpG 1 and 2 by 90% and 70%, respectively (Fig. 3B). Likewise, *in vitro* methylation of the second insert containing CpGs 3 to 10 resulted in increased methylation of CpGs 4 to 9 ranging from 40 to 90%, respectively (Fig. 3B). Subsequent reporter assays in transfected HeLa cells showed a significant decrease in luciferase activity by 40% of the construct containing methylated CpGs 1 and 2 while the construct with methylated CpG sites 4 to 9 showed a weak but non-significant reduction by 20% in luciferase activity (Fig. 3C). These results indicate that methylation of the *SNCA* intron 1 subregion containing CpGs 1 and 2 plays an important role in the repression of the *SNCA* gene expression.

### Mutations Closed to the CpG Sites 1 and 2 Affect SNCA Gene Expression

**Fig. 4:**
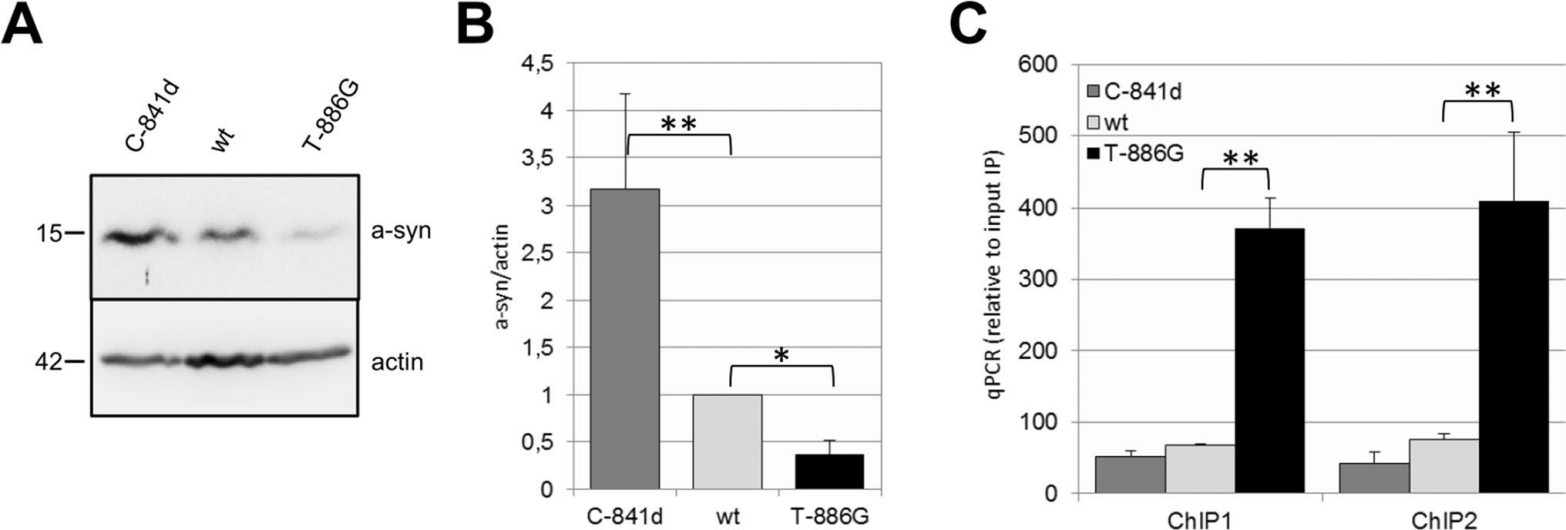
CRISPR/Cas9-mediated mutations in intron 1 of *SNCA* affect the expression of a-syn and binding of MeCP2. **A** Representative Western blot of a-syn and actin using lysates of the wild type (wt) and mutated SK-N-SH cell lines, C-841d and T-886G. **B** Quantification of three individual Western blots by densitometry. Relative protein amounts of a-syn were normalized to actin and compared to untreated SK-N-SH (wt). **C** Enrichment of MeCP2 to *SNCA*-fragments ChIP1 and ChIP2 using chromatin from cell lines C-841d and T-886G by ChIP analysis. Data are presented as means ± s.d. of three independent ChIP reactions. (*) *p* ≤ 0.05; (**) *p* ≤ 0.01

To further assess specific effects of the methylated CpG sites in *SNCA* intron 1 on the transcriptional regulation of the SNCA gene, two guide RNAs targeting two regions adjacent to CpGs 1 and 2 were selected to induce localized mutations generated by on-target Cas9 cutting and repair. SK-N-SH cells were stably co-transfected with CRISPR/Cas9 and one of each gRNAs (gRNA-CpG1 and gRNA-CpG2) targeting CpG1 or CpG2 in the *SNCA* intron 1 (Table 1). Several individual SK-N-SH clonal cell lines were selected and analyzed for a-syn expression by Western blot analysis. Interestingly, gRNA-CpG1 edited cells showed strongly reduced expression levels of a-syn whereas gRNA-CpG2 edited cells revealed increased expression of a-syn (Fig. 4A, B). Sequence analysis of gRNA-CpG1 edited cells revealed a T to G transversion at position − 886 (T-886G) generating a novel CpG site closed to CpG1 and a single nucleotide deletion of a C at position − 841 (C-841d) for the gRNA-CpG2 edited cells (Supplementary Fig. S1). SK-N-SH cells exhibiting the additional CpG site (T-886G) showed a significantly enriched binding of MeCP2 in both *SNCA* intron 1 subregions, ChIP1 and ChIP2, compared to SK-N-SH wild-type cells (Fig. 4C, *p* = 0.0005 and *p* = 0.008). In contrast, SK-N-SH cells with the C deletion (C-841d) showed no MeCP2 enrichment to either *SNCA* intron 1 subregion but a 3.2-fold increase in a-syn levels (Fig. 4A, B). Thus, increased binding of MeCP2 to intron 1 is associated with decreased a-syn expression whereas loss of MeCP2 binding increases expression of a-syn. These findings strongly suggest that transcriptional regulation of *SNCA* expression is controlled by binding of MeCP2 to methylated CpG sites in the *SNCA* intron 1 subregion.

### Knockout and Mutations of MeCP2 Alter A-syn Expression

**Fig. 5:**
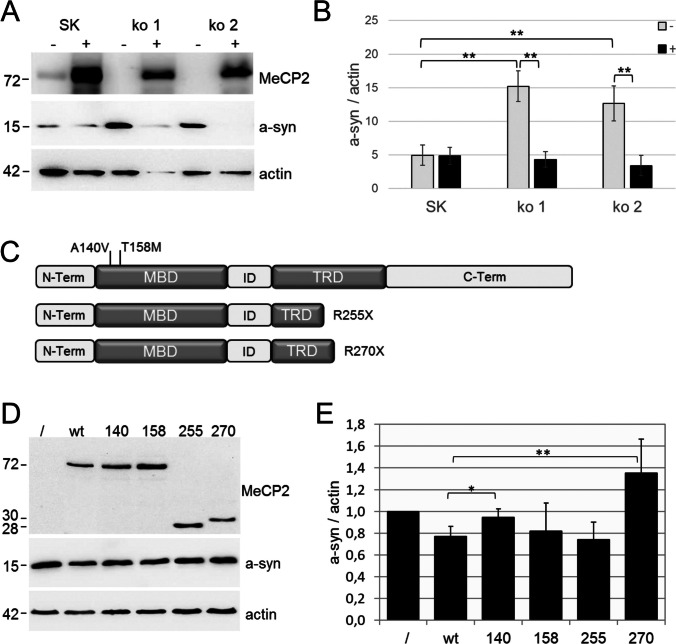
Effects of *MeCP2* knockout and protein variants on a-syn expression. **A** Western blot analysis of MeCP2, a-syn and actin in lysates from native SK-N-SH cells (SK) and MeCP2 knockout cell lines (ko 1 and ko 2) transfected without/with (±) pCMV3-*MeCP2*. **B** Densitometric quantification of a-syn protein levels normalized to actin. **C** Schematic of the MeCP2 domain structure and localization of analyzed MeCP2 mutations. MBD, methyl-CpG binding domain; ID, intervening domain; TRD, transcriptional repression domain. **D** Western blot analysis of MeCP2, a-syn and actin in lysates from MeCP2 knockout cells either not transfected (/) or transfected with wildtype MeCP2 and different MeCP2 variants (wt and variants 140, 158, 255, and 270). **E** Densitometric quantification of a-syn and MeCP2 protein levels normalized to actin in comparison to untransfected *MeCP2* knockout cells (/). Data are presented as means ± s.d. from five independent transfections. (*) *p* ≤ 0.05; (**) *p* ≤ 0.01

To assess the role of MeCP2 in the regulation of *SNCA* transcription, we generated *MeCP2*-knockout cell lines in SK-N-SH cells. To this end, SK-N-SH cells were stably transfected with a CRISPR/Cas9 expression vector and gRNAs targeting exon 2, 3, and 4 of the *MeCP2* gene. Single-cell colonies were selected and analyzed for MeCP2 expression by Western blot analysis. Two stable *MeCP2*-knockout clonal cell lines (ko1 and ko2) edited with the gRNA targeting exon 4 (MeCP2-TS1) showed a complete loss of MeCP2 expression (Fig. 5A). Strikingly, the loss of MeCP2 induced a threefold increase in a-syn levels compared to wild-type SK-N-SH cells (*p* = 0.01). Vice versa, overexpression of recombinant MeCP2 in the knockout cell lines significantly reduced expression levels of a-syn (*p* = 0.01) but not in wild-type SK-N-SH (*p* = 0.84) cells (Fig. 5A, B). Thus, MeCP2 associates with the regulation of *SNCA*.

Several mutations in the *MeCP2* gene have already been described in RTT patients with Parkinson’s features. Therefore, we reasoned whether these mutant variants of *MeCP2* might alter *SNCA* expression. In particular, we were interested to study the effects of the mutation A140D in the methyl-binding domain (MBD), R270X in transcription repression domain (TRD), and the most common RTT mutations T158M in MBD and R255X in TRD [27, 28]. Mutations A140D and T158M are amino acid substitutions, whereas the mutations R255X and R270X result in truncated variants of MeCP2 (Fig. 5C). Expression of wild-type MeCP2 protein in *MeCP2* knockout cells reduced the a-syn levels on average by 23% compared to untransfected cells (Fig. 5D, E). Compared to wild-type MeCP2, expression of the MeCP2 variants T158M and R255X in *MeCP2* knockout cells did not reduce endogenous a-syn expression levels. In contrast, expression of the MeCP2 variant A140D significantly increased the a-syn protein levels compared to *MeCP2* knockout cells transfected with the wild-type MeCP2 protein (*p* = 0.02). By far the strongest induction of a-syn expression was observed with the truncated MeCP2 variant R270X (*p* = 0.007). These findings show that both knockout of the endogenous *MeCP2* gene and expression of two RTT-related protein variants of MeCP2 (A140D, R270X) are associated with increased expression of a-syn. Thus, MeCP2 is most likely involved in the regulation of *SNCA* expression and, moreover, two MeCP2 variants might be associated with PD features observed in RTT patients.

### MeCP2 Variants Show Different Binding Properties to SNCA Intron 1

The previous findings raised the question of whether different binding properties of the MeCP2 protein variants to their DNA motifs could cause altered expression of a-syn. For this purpose, we performed electrophoretic mobility shift assays (EMSA) with unmethylated and methylated *SNCA* intron 1 probes of both CpG1 and CpG2 sites containing the putative MeCP2 binding sites. Formation of high molecular shift complexes was observed only with methylated probes of both CpG1 and CpG2 in agreement with our ChIP findings (Fig. 2B). However, only the wild-type MeCP2 protein and the R255X variant showed increased binding to the methylated CpG1 and CpG2 probes containing the putative MeCP2 binding sites (Supplementary Fig. S2A-B).

**Fig. 6:**
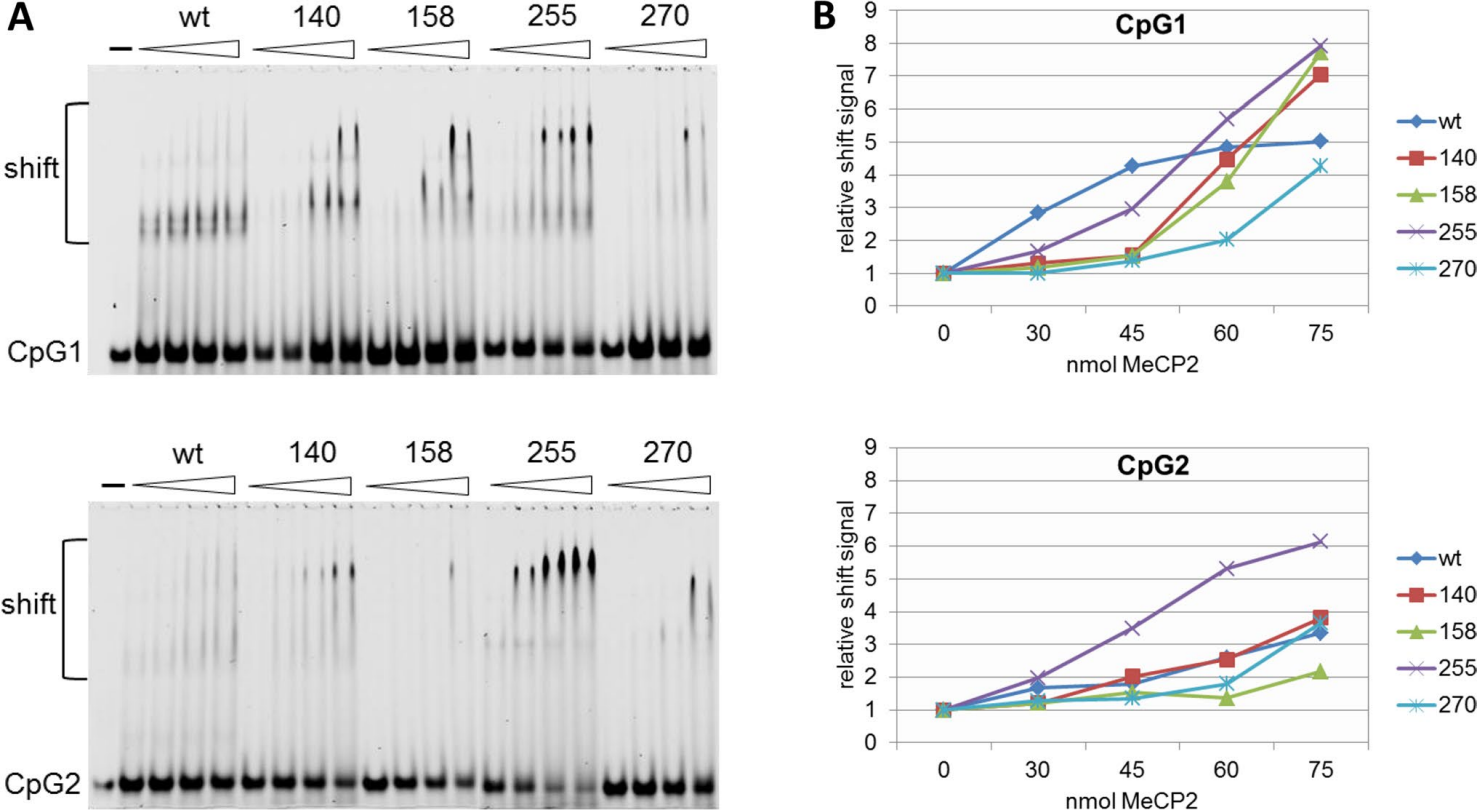
Binding properties of MeCP2 variants to CpG1 and CpG2 in *SNCA* intron 1. **A** EMSAs analyzing the binding to methylated *SNCA* probes CpG1 and CpG2 with increasing protein amounts of MeCP2 (30-75 nmol). (-) indicates free probe. **B** Densitometric quantification of signal intensities. Data are presented as relative shift signal normalized to the signal obtained without protein

To study the binding affinities of the variants in more detail, increasing amounts of the MeCP2 wild-type protein and variants were used and analyzed for their binding to the methylated *SNCA* intron 1 probes of CpG1 and CpG2. At 30 nM, the wild-type MeCP2 and R255X variant already produced detectable band shifts with the CpG1 probe while variants A140D and T158M did not show formation of shift complexes until 50 nM (Fig. 6A, B). The weakest shift complexes were obtained with the R270X variant and only at the highest concentration of 75 nM. Similarly, to the result with the CpG1 probe, variant R255X generated the strongest shift complexes already at 30 nM with the methylated CpG2 probe while the other variants and also the wild-type protein formed only weak or almost undetectable shift complexes with the CpG2 probe (Fig. 6A, B). However, although the R255X variant showed strong binding to the methylated CpG1 and CpG2 probes, this binding apparently does not alter the a-syn expression levels since the a-syn levels were comparable to those of *MeCP2* knockout cells transfected with wild-type MeCP2 (Fig. 5E). Among all variants studied, the R270X variant showed the weakest formation of shift complexes with both the CpG1 and CpG2 probes (Fig. 6). These findings indicate that the R270X variant has probably lost its ability to bind to methylated CpGs in *SNCA* intron 1 resulting in increased expression of a-syn (Fig. 5E).

## Discussion

In this study, we found that MeCP2 binds to methylated CpG1 and CpG2 sites in *SCNA* intron 1 and regulates expression of a-syn protein levels. Interestingly, reduction of MeCP2 levels as well as expression of mutant MeCP2 RTT variants increased a-syn expression. The increased a-syn expression in *MeCP2* knockout cell lines suggest an important role of MeCP2 in the regulation of *SNCA* expression and may indicate a loss of function in some RTT-related variants of *MeCP2*.

*In vitro* studies suggested that MeCP2 can bind both methylated and non-methylated DNA sequences [48]. However, in neuronal cells, where MeCP2 is highly abundant, it associates preferentially with methylated regions [49]. For the methylation-dependent interaction, MeCP2 has been shown to require a methylated CpG and an adjacent [A/T]_4_ motif for high-affinity DNA binding [22]; five such potential CpG sites (CpGs 1, 2, 8, 9, and 10) reside within *SNCA*_(-926/-483)_. This region contains 23 CpGs and is hypomethylated in sPD patients’ brains [10]. Our ChIP experiments confirmed methylation-specific binding of MeCP2 at the analyzed CpGs in SK-N-SH cells. These cells showed strong methylation (~ 70%) at *SNCA* intron 1 while treatment with Aza effectively promoted demethylation (~ 10%). The significant decrease in methylation by Aza resulted in significant reduction of MeCP2 binding. The differences of *SNCA* intron 1 methylation in human brain tissues were much smaller—likewise was the reduction of MeCP2 enrichment in the corresponding ChIP experiments.

DNA methylation represses transcription *in vivo* [50], and MeCP2 binding mediates silencing of gene expression [51]. Differential binding of MeCP2 with downregulation of gene expression was observed after site-specific methylation of the *arginine vasopressin* (*Avp*) gene in mice [44]. We thus tested the impact of individual CpG sites on *SNCA* expression. Specific methylation of CpGs 1 and 2 in the putative binding site of MeCP2 in *SNCA* intron 1 was sufficient to significantly reduce expression of a reporter gene. *In vitro* methylation of CpGs 4-9 was less effective (68% compared to 80% for CpGs 1 and 2), and we therefore conclude that methylation of CpGs 1 and 2 in *SNCA* intron 1 adjacent to MeCP2 binding sites is important for the modulation of *SNCA* gene transcription. To evaluate whether DNA sequence variations also play a role for binding of MeCP2, we introduced localized mutations by CRISPR/Cas9-mediated editing in the immediate vicinity of CpGs 1 and 2. Interestingly, the deletion of a C (C-841d) near the CpG2 site in one of the edited cell lines created novel putative transcription factor (TF) binding sites (Supplementary Fig. S3A) for the glucocorticoid receptor (GR-alpha) [52], cellular E26 transformation-specific transcription factor 2 (c-Ets-2) [53], the general transcription factor II-1 (TFII-1) [54], nuclear factor-kappa B (NF-kappaB) [55], and the enkephalin transcription factor-1 (ENKF-1) [56], all of which have been described to activate expression. Thus, the novel TF binding properties created by deletion of C-841 may also be associated with an increase in a-syn expression.

Interestingly, CRISPR/Cas9-mediated editing of the CpG1 site (T-886G) created a new CpG site and thereby increasing the potential for further DNA methylation. Substitution of T to G at position -886 (near CpG1) increased overall methylation of *SNCA*_(-926/-483)_ (Supplementary Fig. S3B). Rube et al. suggested that MeCP2’s association with methylation is in part due to its affinity to GC-rich chromatin [25]. Together with increased methylation of subsequent CpGs 3-7, this resulted in enhanced binding of MeCP2 at ChIP1. Increased methylation of the CpGs covered by ChIP2 (CpGs 6-13) most likely accounts for the enhanced MeCP2 binding at ChIP2. The observation that a single intronic nucleotide substitution in the human *SNCA* gene alters the methylation of neighboring CpGs, binding MeCP2 and subsequent a-syn production might have implications for other DNA variations in intronic regions. The increased binding of MeCP2 and the subsequently reduced a-syn levels corroborate the importance of *SNCA* intron 1 for transcriptional regulation of *SNCA*. In this regard, it is important to note that hypermethylation following *in vitro* mutation cannot be detected with microarray-based [57, 58] or gene-specific approaches [59, 60] which cannot distinguish between the influence of the analyzed SNPs and other sequence differences in linkage disequilibrium with these SNPs.

While other polymorphisms outside of *SNCA* and associated with PD have also been shown to be significantly associated with both methylation and expression changes [61], methylation studies have yet to examine SNPs associated with PD and located within the *SNCA*_(-926/-483)_ region (upstream of CpG1, between CpG9 and 10 and at CpG19, respectively: rs2619361, rs1372520 and rs3756063).

The role of MeCP2 in *SNCA* expression regulation was additionally demonstrated by knocking out *MeCP2*. Lack of MeCP2 resulted in increased a-syn protein expression which could be reversed by recombinant expression of MeCP2. Loss-of-function mutations of *MeCP2* are associated with RTT in females and with syndromic and non-syndromic forms of mental retardation in males [31].

Restoration of *MeCP2* knockout cells with mutated MeCP2 had different effects. MeCP2-T158M and MeCP2-R255X, which are among the most common mutations in RTT, acted similar to wild-type MECP2 protein. However, expression of MeCP2-A140D and MeCP2-R270X resulted in increased a-syn expression. Reduced binding of these variants to CpG1 was detected by EMSA pointing to the importance of MeCP2 binding to CpG1 for a-syn expression. MeCP2-A140D and MeCP2-R270X are among the mutations in RTT syndrome with parkinsonian symptoms [30]. Resembling PD in the adult, parkinsonian features in RTT appear as late features during progression of RTT—suggesting that a minor increase of a-syn over time is sufficient to trigger the typical symptoms during aging.

Although numerous mouse models with various *Mecp2* mutations (i.e. A140V, T158M and R255X) have been established [62], none of the models has revealed an increase in a-syn expression. This is likely due to the distinct differences of the sequences in the intron 1 region of *SNCA* between humans and mice. CpG1 and CpG2 in particular are not conserved in the mouse sequence, and *Mecp2*-related regulation of a-syn expression in mice may thus differ from humans. Unfortunately, when genes regulated by MeCP2 were analyzed in five RTT-derived induced pluripotent stem cell lines, only the T158M mutation, which in our hands showed no difference to wild type with regard to *SNCA* expression, was included and showed no change, too [63].

## Conclusions

We identified the methylation of *SNCA* intron 1 as an important factor for binding of MeCP2 and the regulation of a-syn expression. The finding that a single nucleotide substitution next to the CpG1 site in the human *SNCA* gene altered adjacent CpG methylation, binding of MeCP2 and a-syn production could be of importance for other PD-related polymorphisms. The observation that a single intronic nucleotide substitution in the human *SNCA* gene alters the methylation of neighboring CpGs, binding MeCP2 and subsequent a-syn production might have implications for other DNA variations in intronic regions.

Altered DNA binding properties of specific MeCP2 variants increased a-syn expression, implicating dysregulated expression of *SNCA* in the pathology of these RTT genotypes.

## Supplementary information

Supplementary file 1

## Data Availability

The data and materials are available from the corresponding author on reasonable request.

## Abbreviations

a-syn/*SNCA*: Alpha-synuclein
Aza: Deoxycytidine analog 5-aza-2’-deoxycytidine
ChIP: Chromatin immunoprecipitation
DN: Dopaminergic neurons
EMSA: Electrophoretic mobility shift assay
ko: Knockout
MBD: Methyl-CpG-binding domain
MeCP2: Methyl-CpG binding protein 2
qPCR: Quantitative PCR
(s)PD: (sporadic) Parkinson’s disease
RTT: Rett syndrome
TF: Transcription factor
TH: Tyrosine hydroxylase
TRD: Transcriptional repression domain
Wt: Wild type

## References

[1] Perez RG (2020) Editorial: The protein alpha-synuclein: its normal role (in neurons) and its role in disease. Front Neurosci 14:116 32153354 10.3389/fnins.2020.00116 PMC7044239

[2] Spillantini MG, Schmidt ML, Lee VM, Trojanowski JQ, Jakes R, Goedert M (1997) Alpha-synuclein in Lewy bodies. Nature. 388:839–840 9278044 10.1038/42166

[3] Braak H, Del TK, Rub U, de Vos RA, Jansen Steur EN, Braak E (2003) Staging of brain pathology related to sporadic Parkinson’s disease. Neurobiol Aging 24:197–211 12498954 10.1016/s0197-4580(02)00065-9

[4] Hernandez DG, Reed X, Singleton AB (2016) Genetics in Parkinson disease: Mendelian versus non-Mendelian inheritance. J Neurochem 139(Suppl 1):59–74 27090875 10.1111/jnc.13593 PMC5155439

[5] Yang JO, Kim W-Y, Jeong S-Y, Oh J-H, Jho S, Bhak J, Kim N-S (2009) PDbase: a database of Parkinson’s disease-related genes and genetic variation using substantia nigra ESTs. BMC Genomics 10:S32 http://bioportal.kobic.re.kr/PDbase/;http://bioportal.kobic.re.kr/PDbase/suppl.jsp 19958497 10.1186/1471-2164-10-S3-S32 PMC2788386

[6] Holzmann C, Krüger R, Saecker AM, Schmitt I, Schöls L, Berger K, Riess O (2003) Polymorphisms of the alpha-synuclein promoter: expression analyses and association studies in Parkinson’s disease. J Neural Transm 110(1):67–76 12541013 10.1007/s00702-002-0769-5

[7] GEO-PD Consortium (2006) Collaborative analysis of alpha-synuclein gene promoter variability and Parkinson disease. JAMA 296(6):661–670 16896109 10.1001/jama.296.6.661

[8] Grundemann J, Schlaudraff F, Haeckel O, Liss B (2008) Elevated alpha-synuclein mRNA levels in individual UV-laser-microdissected dopaminergic substantia nigra neurons in idiopathic Parkinson’s disease. Nucleic Acids Res 36:e38 18332041 10.1093/nar/gkn084 PMC2367701

[9] Cheng F, Zheng W, Liu C, Barbuti PA, Yu-Taeger L, Casadei N, Huebener-Schmid J, Admard J, Boldt K, Junger K, Ueffing M, Houlden H, Sharma M, Kruger R, Grundmann-Hauser K, Ott T, Riess O (2022) Intronic enhancers of the human SNCA gene predominantly regulate its expression in brain in vivo. Sci Adv 8(47):eabq6324. 10.1126/sciadv.abq6324 10.1126/sciadv.abq6324 PMC9683720 36417521

[10] Clough RL, Stefanis L (2007) A novel pathway for transcriptional regulation of alpha-synuclein. FASEB J 21:596–607 17167067 10.1096/fj.06-7111com

[11] Scherzer CR, Grass JA, Liao Z et al (2008) GATA transcription factors directly regulate the Parkinson’s disease-linked gene alpha-synuclein. Proc Natl Acad Sci USA 105:10907–10912 18669654 10.1073/pnas.0802437105 PMC2504800

[12] Brenner S, Wersinger C, Gasser T (2015) Transcriptional regulation of the α-synuclein gene in human brain tissue. Neurosci Lett 99:140–145 10.1016/j.neulet.2015.05.029 26002080

[13] Jowaed A, Schmitt I, Kaut O, Wullner U (2010) Methylation regulates alpha-synuclein expression and is decreased in Parkinson’s disease patients’ brains. J Neurosci 30:6355–6359 20445061 10.1523/JNEUROSCI.6119-09.2010 PMC6632710

[14] Kantor B, Tagliafierro L, Gu J et al (2018) Downregulation of SNCA expression by targeted editing of DNA methylation: a potential strategy for precision therapy in PD. Mol Ther 26(11):2638–2649 30266652 10.1016/j.ymthe.2018.08.019 PMC6224806

[15] Xie J, Xie L, Wei H, Li XJ, Lin L (2023) Dynamic regulation of DNA methylation and brain functions. Biology (Basel) 12(2):152. 10.3390/biology12020152 36829430 10.3390/biology12020152 PMC9952911

[16] Hendrich B, Bird A (1998) Identification and characterization of a family of mammalian methyl-CpG binding proteins. Mol Cell Biol 18(11):6538–6547 9774669 10.1128/mcb.18.11.6538 PMC109239

[17] Clouaire T, Stancheva I (2008) Methyl-CpG binding proteins: specialized transcriptional repressors or structural components of chromatin? Cell Mol Life Sci 65(10):1509–1522 18322651 10.1007/s00018-008-7324-y PMC2873564

[18] Medvedeva YA, Khamis AM, Kulakovskiy IV et al (2014) Effects of cytosine methylation on transcription factor binding sites. BMC Genomics 15:119 24669864 10.1186/1471-2164-15-119 PMC3986887

[19] Luikenhuis S, Giacometti E, Beard CF, Jaenisch R (2004) Expression of MeCP2 in postmitotic neurons rescues Rett syndrome in mice. Proc Natl Acad Sci USA 101:6033–6038 15069197 10.1073/pnas.0401626101 PMC395918

[20] Yasui DH, Peddada S, Bieda MC et al (2007) Integrated epigenomic analyses of neuronal MeCP2 reveal a role for long-range interaction with active genes. Proc Natl Acad Sci USA 104:19416–19421 18042715 10.1073/pnas.0707442104 PMC2148304

[21] Nan X, Tate P, Li E, Bird A (1996) DNA methylation specifies chromosomal localization of MeCP2. Mol Cell Biol 16(1):414–421 8524323 10.1128/mcb.16.1.414 PMC231017

[22] Klose RJ, Sarraf SA, Schmiedeberg L, McDermott SM, Stancheva I, Bird AP (2005) DNA binding selectivity of MeCP2 due to a requirement for A/T sequences adjacent to methyl-CpG. Mol Cell 19:667–678 16137622 10.1016/j.molcel.2005.07.021

[23] Ho KL, McNae IW, Schmiedeberg L, Klose RJ, Bird AP, Walkinshaw MD (2008) MeCP2 binding to DNA depends upon hydration at methyl-CpG. Mol Cell 29(4):525–531 18313390 10.1016/j.molcel.2007.12.028

[24] Chen L, Chen K, Lavery LA, Baker SA, Shaw CA, Li W, Zoghbi HY (2015) MeCP2 binds to non-CG methylated DNA as neurons mature, influencing transcription and the timing of onset for Rett syndrome. Proc Natl Acad Sci USA 112(17):5509–5514 25870282 10.1073/pnas.1505909112 PMC4418849

[25] Rube HT, Lee W, Hejna M et al (2016) Sequence features accurately predict genome-wide MeCP2 binding in vivo. Nat Commun 7:11025 27008915 10.1038/ncomms11025 PMC4820824

[26] Rett A (1986) Rett syndrome. History and general overview. Am J Med Genet Suppl 1:21–25 10.1002/ajmg.1320250503 3087183

[27] Amir RE, Van den Veyver IB, Wan M, Tran CQ, Francke U, Zoghbi HY (1999) Rett syndrome is caused by mutations in X-linked MECP2, encoding methyl-CpG-binding protein 2. Nat Genet 23:185–188 10508514 10.1038/13810

[28] Smeets E, Schollen E, Moog U et al (2003) Rett syndrome in adolescent and adult females: clinical and molecular genetic findings. Am J Med Genet A 122A(3):227–233 12966523 10.1002/ajmg.a.20321

[29] Villard L (2007) MECP2 mutations in males. J Med Genet 44(7):417–423 17351020 10.1136/jmg.2007.049452 PMC2597995

[30] Weaving LS, Williamson SL, Bennetts B et al (2003) Effects of MECP2 mutation type, location and X-inactivation in modulating Rett syndrome phenotype. Am J Med Genet A 118A(2):103–114 12655490 10.1002/ajmg.a.10053

[31] Chahrour M, Zoghbi HY (2007) The story of Rett syndrome: from clinic to neurobiology. Neuron. 563:422–437 10.1016/j.neuron.2007.10.001 17988628

[32] Lekman A, Witt-Engerström I, Gottfries J, Hagberg BA, Percy AK, Svennerholm L (1989) Rett syndrome: biogenic amines and metabolites in postmortem brain. Pediatr Neurol 5(6):357–362 2604799 10.1016/0887-8994(89)90049-0

[33] Wenk GL, Naidu S, Casanova MF, Kitt CA, Moser H (1991) Altered neurochemical markers in Rett’s syndrome. Neurology. 41(11):1753–1756 1658685 10.1212/wnl.41.11.1753

[34] FitzGerald PM, Jankovic J, Percy AK (1990) Rett syndrome and associated movement disorders. Mov Disord 5(3):195–202 2388636 10.1002/mds.870050303

[35] Riederer P, Weiser M, Wichart I, Schmidt B, Killian W, Rett A (1986) Preliminary brain autopsy findings in progredient Rett syndrome. Am J Med Genet Suppl 1:305–315 10.1002/ajmg.1320250530 3087191

[36] Chiron C, Bulteau C, Loc'h C, Raynaud C, Garreau B, Syrota A, Mazière B (1993) Dopaminergic D2 receptor SPECT imaging in Rett syndrome: increase of specific binding in striatum. J Nucl Med 34(10):1717–1721 8410289

[37] Kitt CA, Wilcox BJ (1995) Preliminary evidence for neurodegenerative changes in the substantia nigra of Rett syndrome. Neuropediatrics. 26(2):114–118 7566448 10.1055/s-2007-979739

[38] Jellinger K, Armstrong D, Zoghbi HY, Percy AK (1988) Neuropathology of Rett syndrome. Acta Neuropathol 76(2):142–158 2900587 10.1007/BF00688098

[39] Jellinger KA (2003) Rett Syndrome -- an update. J Neural Transm (Vienna) 110(6):681–701 12768363 10.1007/s00702-003-0822-z

[40] Panayotis N, Pratte M, Borges-Correia A, Ghata A, Villard L, Roux JC (2011) Morphological and functional alterations in the substantia nigra pars compacta of the Mecp2-null mouse. Neurobiol Dis 41(2):385–397 20951208 10.1016/j.nbd.2010.10.006

[41] Szczesna K, de la Caridad O, Petazzi P et al (2014) Improvement of the Rett syndrome phenotype in a MeCP2 mouse model upon treatment with levodopa and a dopa-decarboxylase inhibitor. Neuropsychopharmacology. 39(12):2846–2856 24917201 10.1038/npp.2014.136 PMC4200495

[42] Ide S, Itoh M, Goto Y (2005) Defect in normal developmental increase of the brain biogenic amine concentrations in the mecp2-null mouse. Neurosci Lett 386(1):14–17 15975715 10.1016/j.neulet.2005.05.056

[43] Viemari JC, Roux JC, Tryba AK et al (2005) Mecp2 deficiency disrupts norepinephrine and respiratory systems in mice. J Neurosci 25(50):11521–11530 16354910 10.1523/JNEUROSCI.4373-05.2005 PMC6726028

[44] Murgatroyd C, Patchev AV, Wu Y et al (2009) Dynamic DNA methylation programs persistent adverse effects of early-life stress. Nat Neurosci 12:1559–1566 19898468 10.1038/nn.2436

[45] Gossen M, Bujard H (1992) Tight control of gene expression in mammalian cells by tetracycline-responsive promoters. Proc Natl Acad Sci USA 89(12):5547–5551 1319065 10.1073/pnas.89.12.5547 PMC49329

[46] Jullien N, Herman JP (2011) LUEGO: a cost and time saving gel shift procedure. Biotechniques. 51(4):267–269 21988693 10.2144/000113751

[47] Matsumoto L, Takuma H, Tamaoka A et al (2010) CpG demethylation enhances alpha-synuclein expression and affects the pathogenesis of Parkinson’s disease. PLoS One 5(11):e15522 21124796 10.1371/journal.pone.0015522 PMC2991358

[48] Nikitina T, Shi X, Ghosh RP, Horowitz-Scherer RA, Hansen JC, Woodcock CL (2007) Multiple modes of interaction between the methylated DNA binding protein MeCP2 and chromatin. Mol Cell Biol 27:864–877 17101771 10.1128/MCB.01593-06 PMC1800686

[49] Skene PJ, Illingworth RS, Webb S et al (2010) Neuronal MeCP2 is expressed at near histone-octamer levels and globally alters the chromatin state. Mol Cell 37:457–468 20188665 10.1016/j.molcel.2010.01.030 PMC4338610

[50] Siegfried Z, Eden S, Mendelsohn M, Feng X, Tsuberi BZ, Cedar H (1999) DNA methylation represses transcription in vivo. Nat Genet 22:203–206 10369268 10.1038/9727

[51] Nan X, Ng HH, Johnson CA, Laherty CD, Turner BM, Eisenman RN, Bird A (1998) Transcriptional repression by the methyl-CpG-binding protein MeCP2 involves a histone deacetylase complex. Nature. 393:386–389 9620804 10.1038/30764

[52] Burd CJ, Archer TK (2013) Chromatin architecture defines the glucocorticoid response. Mol Cell Endocrinol 380(1-2):25–31 23545159 10.1016/j.mce.2013.03.020 PMC3762934

[53] Basuyaux JP, Ferreira E, Stéhelin D, Butticè G (1997) The Ets transcription factors interact with each other and with the c-Fos/c-Jun complex via distinct protein domains in a DNA-dependent and -independent manner. J Biol Chem 272(42):26188–26195 9334186 10.1074/jbc.272.42.26188

[54] Roy AL (2001) Biochemistry and biology of the inducible multifunctional transcription factor TFII-I. Gene. 274(1-2):1–13 11674993 10.1016/s0378-1119(01)00625-4

[55] Meffert MK, Chang JM, Wiltgen BJ, Fanselow MS, Baltimore D (2003) NF-kappa B functions in synaptic signaling and behavior [published correction appears in Nat Neurosci. 2003 Dec;6(12):1329]. Nat Neurosci 6(10):1072–1078 12947408 10.1038/nn1110

[56] Comb M, Mermod N, Hyman SE, Pearlberg J, Ross ME, Goodman HM (1988) Proteins bound at adjacent DNA elements act synergistically to regulate human proenkephalin cAMP inducible transcription. EMBO J 7(12):3793–3805 2850173 10.1002/j.1460-2075.1988.tb03264.x PMC454956

[57] Kerkel K, Spadola A, Yuan E et al (2008) Genomic surveys by methylation-sensitive SNP analysis identify sequence-dependent allele-specific DNA methylation. Nat Genet 40(7):904–908 18568024 10.1038/ng.174

[58] Hellman A, Chess A (2010) Extensive sequence-influenced DNA methylation polymorphism in the human genome. Epigenetics Chromatin 3(1):11 20497546 10.1186/1756-8935-3-11 PMC2893533

[59] Moser D, Ekawardhani S, Kumsta R et al (2009) Functional analysis of a potassium-chloride co-transporter 3 (SLC12A6) promoter polymorphism leading to an additional DNA methylation site. Neuropsychopharmacology. 34(2):458–467 18536702 10.1038/npp.2008.77

[60] Pathak H, Frieling H, Bleich S et al (2017) Promoter polymorphism rs886205 genotype interacts with DNA methylation of the ALDH2 regulatory region in alcohol dependence. Alcohol Alcohol 52(3):269–276 28430929 10.1093/alcalc/agw106

[61] Nalls MA, Saad M, Noyce AJ et al (2014) Genetic comorbidities in Parkinson’s disease. Hum Mol Genet 23(3):831–841 24057672 10.1093/hmg/ddt465 PMC3888265

[62] Vashi N, Justice MJ (2019) Treating Rett syndrome: from mouse models to human therapies. Mamm Genome 5-6:90–110 10.1007/s00335-019-09793-5 PMC6606665 30820643

[63] Tanaka Y, Kim KY, Zhong M, Pan X, Weissman SM, Park IH (2014) Transcriptional regulation in pluripotent stem cells by methyl CpG-binding protein 2 (MeCP2). Hum Mol Genet 23(4):1045–1055 24129406 10.1093/hmg/ddt500 PMC3900111

