# Supplementary material for: The Alpha-Synuclein Gene (SNCA) is a Genomic Target of Methyl-CpG Binding Protein 2 (MeCP2)—Implications for Parkinson’s Disease and Rett Syndrome
Source: Mol Neurobiol. 2024 Mar 2;61(10):7830–44. doi: 10.1007/s12035-024-03974-3 (PMC11415397; doi:10.1007/s12035-024-03974-3)
Supplement: Supplementary file 1 [file 12035_2024_3974_MOESM1_ESM.docx]

**Supplementary Figures**


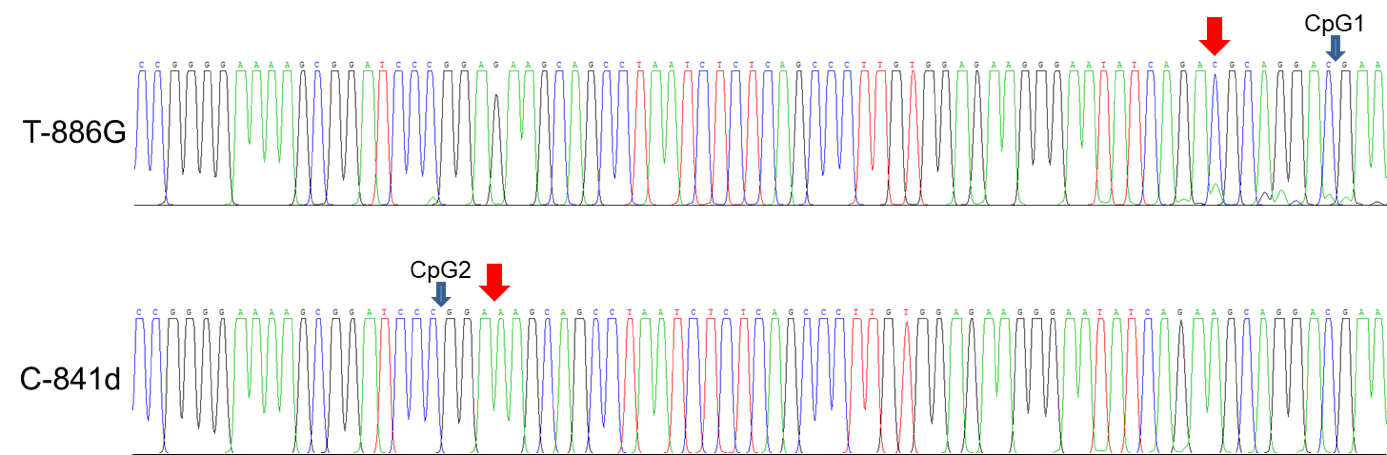


**Supplementary Fig S1.** Sequencing chromatograms (reverse sequence). Substitution of A-886 to C (T-886G) near CpG1 and deletion of G-841 (C-841d) near CpG2 are highlighted by arrows.

**
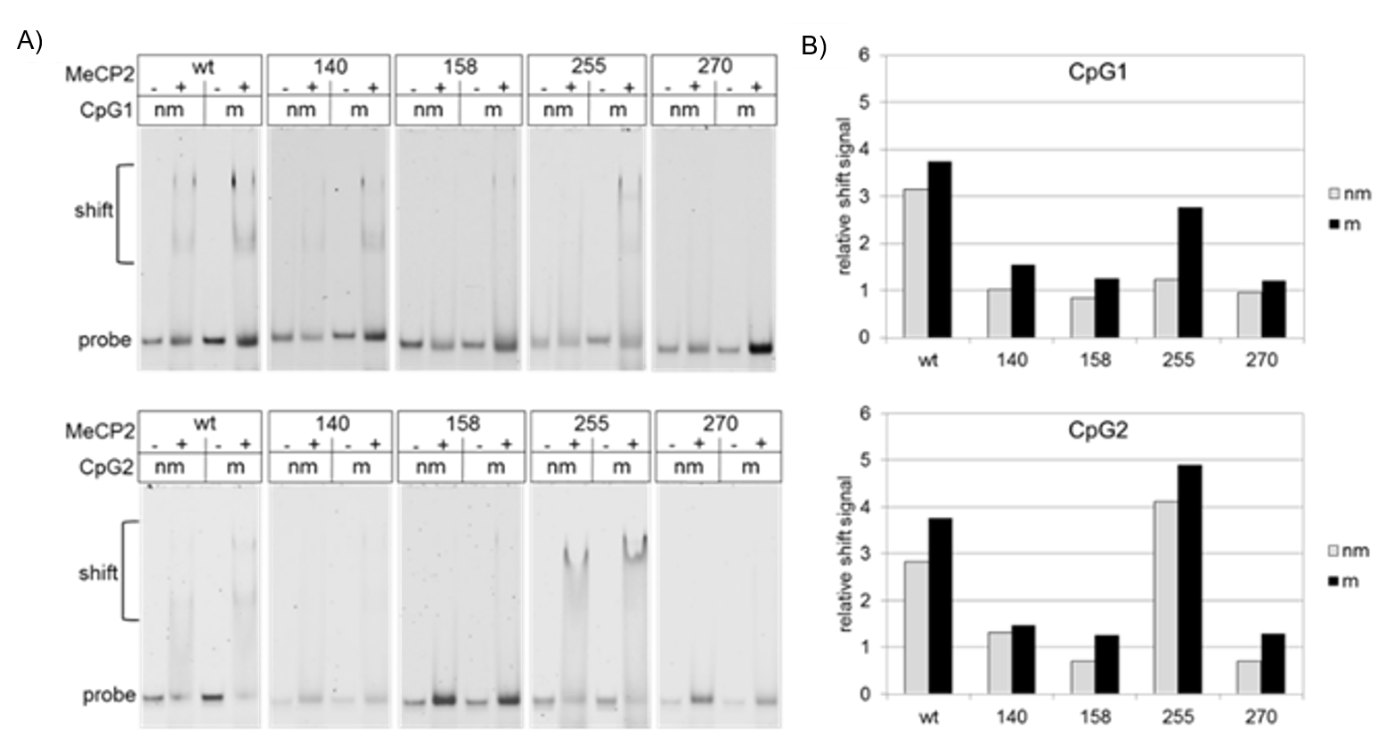
**

**Supplementary Fig** **S2.** Binding studies of wildtype (wt) and mutated MeCP2 variants (A140D, T158M, R255X and R270X) to CpG1 and CpG2 in SNCA intron 1 using EMSAs. A) Non-methylated (nm) or *in vitro* methylated (m), fluorescent-labeled oligonucleotide probes containing CpG1 and CpG2 sites were each incubated without (-) or with 50 nmol MeCP2 protein (+) and separated in non-denaturing polyacrylamide gels. DNA-protein shift complexes were predominantly detected with methylated probes only. B) Quantification of signal intensities normalized to the respective intensities obtained without protein.

***~~
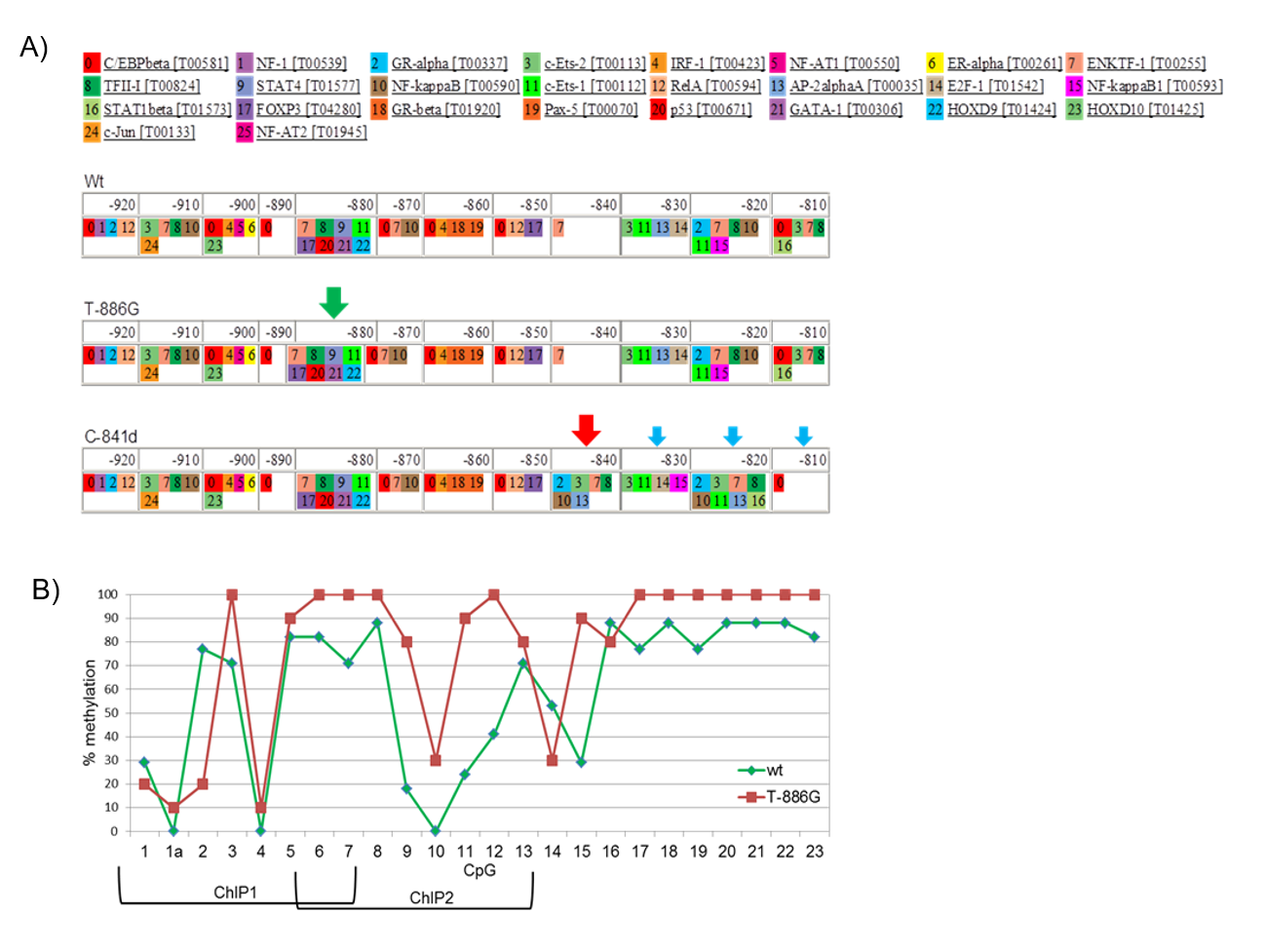
~~***

**Supplementary Fig S3.** A) Prediction of altered transcription factor (TF) binding sites in SNCA intron 1 in the mutant sequence variants of the T-886G and C-841d cell lines compared with the wild-type (wt) sequence. Green arrow: position of the substitution T-886 to G; red arrow: position of the deletion C-841; blue arrows: altered TF binding sites. B) Methylation pattern of adjacent CpGs in wt and T-886G cell lines.
